# Disrupted human–pathogen co-evolution: a model for disease

**DOI:** 10.3389/fgene.2014.00290

**Published:** 2014-08-25

**Authors:** Nuri Kodaman, Rafal S. Sobota, Robertino Mera, Barbara G. Schneider, Scott M. Williams

**Affiliations:** ^1^Department of Genetics, Geisel School of Medicine, Dartmouth CollegeHanover, NH, USA; ^2^Department of Molecular Physiology and Biophysics, Center for Human Genetics Research, Vanderbilt University Medical CenterNashville, TN, USA; ^3^Division of Gastroenterology, Department of Medicine, Vanderbilt University Medical CenterNashville, TN, USA

**Keywords:** host–pathogen co-evolution, human disease, *Helicobacter pylori*, *Mycobacterium tuberculosis*, human papillomavirus, genome–genome interactions

## Abstract

A major goal in infectious disease research is to identify the human and pathogenic genetic variants that explain differences in microbial pathogenesis. However, neither pathogenic strain nor human genetic variation in isolation has proven adequate to explain the heterogeneity of disease pathology. We suggest that disrupted co-evolution between a pathogen and its human host can explain variation in disease outcomes, and that genome-by-genome interactions should therefore be incorporated into genetic models of disease caused by infectious agents. Genetic epidemiological studies that fail to take both the pathogen and host into account can lead to false and misleading conclusions about disease etiology. We discuss our model in the context of three pathogens, *Helicobacter pylori*, *Mycobacterium tuberculosis* and human papillomavirus, and generalize the conditions under which it may be applicable.

## INTRODUCTION

Human response to infectious agents is known to be highly heritable, but identifying the genetic variants responsible for differences in disease susceptibility has proven difficult. Pathogenic variation has, in some cases, become a better predictor of disease outcome, but it too does not sufficiently predict whether a given individual or class of individuals will present with disease. Thus far, genetic epidemiological studies of infectious disease have typically sought to explain the inter-individual variation in disease phenotypes by assessing genetic factors in humans or pathogens alone, under the implicit assumption that these factors have effects that are essentially independent of each other. Here, we argue that genome-by-genome interactions between host and pathogen are likely to play a major role in infectious disease etiology, and as such, should be incorporated into genetic epidemiological models. In short, insofar as host and pathogen jointly determine disease phenotypes, no genetic variant in either should be considered harmful without taking the context of the other into account.

The term “interaction” has two related but distinct meanings in the context of infectious disease, one molecular, and one statistical. Here we refer mainly to the statistical meaning of the term. At the individual level, all aspects of pathogenesis involve molecular interactions of varying importance, e.g., between a pathogenic epitope and a host receptor. Such interactions can be detected statistically, however, only when multiple variants exist in a population and when specific pairings lead to different effects. In some cases, pathogenic variants may function independently of host variation, and vice versa. However, because many pathogens have co-existed with their human hosts for millennia and have likely co-evolved with them, we argue here that statistical interactions, where appropriately sought, will often be found, with profound biomedical implications.

Recent advances in genomics have provided both the impetus and the means to evaluate human–pathogen co-evolutionary hypotheses directly. Whole-genome sequencing of many pathogenic species has substantially improved the resolution with which we classify strains, and facilitated the detection of potentially virulent genetic variants. A clearer picture of microbial evolution has also emerged, marked by selective mechanisms such as rapid gene gain/loss and horizontal gene transfer (Pallen and Wren, 2007). Overlaying human genetic variation onto this emerging evolutionary picture of microbial diversity offers the potential to make the pathogenic process more transparent.

The past few decades have also seen an explosion in studies seeking to identify human susceptibility loci for infectious diseases (Rowell et al., 2012). Candidate gene and family based linkage studies have identified several common polymorphisms with clinical significance at the population level, such as the *CCR5* deletion that protects against HIV (Samson et al., 1996; Picard et al., 2006; Casanova and Abel, 2007). However, most human susceptibility is in fact polygenic, with individual polymorphisms conferring small marginal effects (Hill, 2001). Where infectious disease phenotypes deviate from the “one susceptibility locus – one infection” model, elucidating the genetic architecture underlying inter-individual variation has proven elusive.

While genome-wide association studies (GWAS) may be better designed to accommodate multifactorial phenotypes, those performed thus far on infectious diseases have typically been less informative than GWAS performed on complex non-communicable diseases (Jallow et al., 2009; Hill, 2012; Ko and Urban, 2013). A major challenge facing the GWAS of infectious disease has been the recruitment of a sufficient number of cases and matched controls to achieve adequate statistical power (Hill, 2012; Ko and Urban, 2013). Another potential drawback, and the one that concerns us here, is the fact that many infectious disease phenotypes depend on complex interactions between host and pathogen genomes. In such cases, the pooling together of human samples infected with even subtly different pathogenic strains can obscure genetic associations (Hill, 2012; Ko and Urban, 2013). A problem common to all GWAS is that the statistical effect sizes of biologically meaningful polymorphisms are often too small to pass significance thresholds after correction for multiple testing. This problem is exacerbated, however, when human polymorphisms (or networks of polymorphisms) (Wilfert and Schmid-Hempel, 2008) confer variable, or even opposite effects in the context of different pathogenic strains within the same study cohort. In this regard, it is perhaps telling that the most successful GWAS performed on infectious disease susceptibility to date have been on leprosy; the signal-to-noise ratios in these association studies may be higher because *Mycobacterium leprae* exhibits substantially less genetic heterogeneity than many other pathogens (Monot et al., 2009; Hill, 2012).

There is in fact strong empirical and theoretical justification for the hypothesis that the effects of susceptibility and virulence alleles in the respective gene pools of humans and pathogens are often contingent upon each other. The evolution of virulence is a dynamic process, easily perturbed by extrinsic variables over space and time, and therefore unlikely to follow the same trajectory in every population. For example, a spike in the density of hosts available for transmission can select for increased virulence, by reducing the cost of lethal harm (Anderson and May, 1982). If a pathogen is transmitted vertically (parent to child), the genetic factors that affect pathogenicity are “co-inherited” by host and pathogen, often promoting commensalism (Frank, 1996; Messenger et al., 1999). Even in these cases, the adventitious introduction of a microbial competitor can induce a commensal species to evolve a defensive toxin that harms the host, if only incidentally (Blaser and Kirschner, 2007; Frank and Schmid-Hempel, 2008). The evolution of defenses against pathogenic harm must also navigate fitness tradeoffs that vary with population, including tradeoffs pertaining to the correlated nature of complex traits (Lambrechts et al., 2006). As pathogens evolve rapidly, exerting strong selective pressures on different human populations, host phenotypes will respond in the *ad hoc* manner typical of evolution, limited by the available genetic variation at hand (Jacob, 1977). Whether the result is a steady-state equilibrium due to a perpetual “arms race” or a commensal detente, the same genes and pathways are unlikely to be involved in every population. As a consequence, when humans and pathogens migrate to new environments or admix, the ensuing disruption of co-evolutionary equilibria and loss of complementarity between host and pathogen genotypes may yield unpredictable and potentially deleterious biomedical consequences.

Our emphasis on the significance of mismatched traits is consistent with the genetic mosaic theory of co-evolution, which aims to account for why virtually all co-evolutionary interactions observed in natural populations show spatial variation in outcomes (Thompson et al., 2002; Thompson, 2014). The theory posits that co-evolution occurs in the context of geographically distinct “selection mosaics,” each characterized by a unique genetic and environmental profile, where environmental variables can include both biotic and abiotic factors. Every selection mosaic progresses toward its own co-evolutionary equilibrium, while gene flow between selection mosaics ensures that patterns of maladaptation will be common and detectable where properly studied (Thompson et al., 2002; Ridenhour and Nuismer, 2007).

Despite the likely etiological importance of human–pathogen co-evolution, attempts at empirical confirmation have been rare. Indeed, “proof” of co-evolution poses a formidable challenge, requiring a demonstration of increased reproductive fitness in each species driven by reciprocal changes in two genomes over time (Woolhouse et al., 2002). Although these criteria have been met in laboratory studies and in some natural populations (Lenski and Levin, 1985; Little, 2002; Little et al., 2006), a similarly rigorous assessment of human–pathogen co-evolution must accommodate long generation times and the genetic and phenotypic complexity of the human traits under selection. Nonetheless, substantial phenomenological evidence consistent with human–pathogen co-evolution now exists, including evidence of spatial patterns of parallel genetic variation between species, and of correlated functional changes at the molecular level (Kraaijeveld et al., 1998; Lively and Dybdahl, 2000; Funk et al., 2000; Woolhouse et al., 2002). The collection of high-density genomic data in paired human–pathogen samples and improvements in phenotypic data, as well as advances in pathogen genomics, should soon enable more explicit tests of the concept.

Our aim here is to summarize the growing body of evidence in favor of the hypothesis that genetic interactions driven by host and pathogen co-evolution can have significant implications for genetic epidemiological studies and biomedicine. While this is not a novel hypothesis, it remains understudied. We also underscore how recent advances in genomic technology provide new opportunities to test for genome-by-genome interactions, and offer suggestions on how to incorporate them into more accurate genetic models of disease.

## HELICOBACTER PYLORI

Studies of *Helicobacter pylori* provide perhaps the best evidence in favor of human–pathogen co-evolution, and distinctly illustrate the power of the modern genetic toolkit to investigate it. *H. pylori* chronically infects the gastric epithelia of half the world’s population, causing peptic ulcers in 10–20% of those infected, and distal gastric carcinoma in ∼1% (Peek and Blaser, 2002; Jemal et al., 2011). The majority of individuals infected, however, suffer only from superficial gastritis in adulthood, while likely gaining protection against diseases such as esophageal cancer and reflux esophagitis, and more controversially, childhood asthma and diarrhea (Rothenbacher et al., 2000; Vaezi et al., 2000; Blaser et al., 2008). That *H. pylori* should have a largely innocuous and potentially symbiotic relationship with its host follows from co-evolutionary theory, based on its vertical mode of transmission, its long-term colonization of a single host, and its ∼50,000 year association with *Homo sapiens* (Rothenbacher et al., 2002; Moodley et al., 2012). Why a fraction of individuals develop life-threatening clinical disease, on the other hand, requires explanation, with one possibility being the disruption of long-standing co-evolutionary relationships.

Although *H. pylori*-mediated diseases often advance to the clinical stage in late adulthood, their onset typically occurs during reproductive years (Correa et al., 1976; Susser and Stein, 2002). Importantly, a disease need not have an especially large selection coefficient to shape allele frequency distributions in populations, especially over thousands of years (Ewald and Cochran, 2000). In fact, the historical fitness load of peptic ulcers, obtained by multiplying prevalence by selection coefficient, has been estimated to be similar to those for infectious diseases such as meningitis and rubella (Cochran et al., 2000). Also consistent with co-evolutionary theory is the fact that *H. pylori*-mediated gastric diseases occur disproportionately in men (Susser and Stein, 2002; Engel et al., 2003); *H. pylori* is usually, but not necessarily, transmitted by the mother, such that female fitness has likely exerted a stronger constraint against *H. pylori* virulence.

Some *H. pylori* virulence factors appear to increase the risk of serious clinical outcome regardless of host genotype. The *cag* pathogenicity island, present in some strains, encodes a type IV secretion system, and *VacA* encodes a pore-forming cytotoxin. Both have been implicated as carcinogenic risk factors, though neither is a necessary nor sufficient one (Wroblewski et al., 2010). Other virulence factors released by *H. pylori* include urease, which facilitates neutralization of the otherwise forbidding acidity of the gastric mucosa; NAP, which enables iron uptake; and arginase, which helps *H. pylori* subvert host macrophages. These, like most *H. pylori* virulence factors, operate to create a basal inflammatory state without generating an excessive immune response. Serious clinical disease reflects a disturbance of this balance (Baldari et al., 2005; Blaser and Kirschner, 2007; Salama et al., 2013).

The maintenance of this balance also depends partly on human genetic factors (Lichtenstein et al., 2000; Chiba et al., 2006; Mayerle et al., 2013a). Candidate gene studies on *H. pylori*-mediated diseases have implicated several gene polymorphisms that appear to affect risk, most notably in the interleukin-1 (IL-1) family of cytokines (Schneider et al., 2008). Recently, two GWAS assessing susceptibility to gastric cancer and *H. pylori* infection identified SNPs with odds ratios ranging from 1.3 to 1.4, mostly of uncertain biological function (Shi et al., 2011; El-Omar, 2013; Mayerle et al., 2013b, **Table 1**). These polymorphisms account for only a small proportion of the estimated heritability of disease phenotypes.

**Table 1:** Genetic variants identified by GWAS for phenotypes related to infection by *H. pylori*, *M. tuberculosis*, and human papillomavirus.

Disease/trait	Gene	SNP	Cases/controls	Population	*p*-value	OR^1^	95% CI^2^	Reference
Gastric cancer	*ZBTB20*	rs9841504	1006/2273	Chinese	1.7E-09	0.76	[0.69–0.83]	Shi et al. (2011)
Gastric cancer	*PRKAA1*	rs13361707	1006/2273	Chinese	7.6E-29	1.41	[1.32–1.49]	Shi et al. (2011)
*H. pylori* serologic status	*TLR10*	rs10004195	2623/7862	European	1.4E-18	0.70	[0.65–0.76]	Mayerle et al. (2013b)
*H. pylori* serologic status	*FCGR2A*	rs368433	2623/7862	European	2.1E-08	0.73	[0.65–0.85]	Mayerle et al. (2013b)
Tuberculosis	*RCN1–WT1*	rs2057178	2127/5636	African	2.6E-09	0.77	[0.71–0.84]	Thye et al. (2012)
Tuberculosis	*RPS4XP18–UBE2CP2*	rs4331426	2237/3122	African	6.8E-09	1.19	[1.13–1.27]	Thye et al. (2010)
Cervical cancer	*EXOC1*	rs13117307	1364/3028	Chinese	9.7E-09	1.26	[1.16–1.36]	Shi et al. (2013)
Cervical cancer	*HLA-DPB2*	rs4282438	1364/3028	Chinese	4.5E-27	0.75	[0.71–0.79]	Shi et al. (2013)
Cervical cancer	*ZPBP2–GSDMB*	rs8067378	1364/3028	Chinese	2.0E-08	1.18	[1.11–1.25]	Shi et al. (2013)
Cervical cancer	*–*	rs9277952	1364/3028	Chinese	2.3E-09	0.85	[0.81–0.90]	Shi et al. (2013)
Cervical cancer	*MICA*	rs2516448	2174/5002	European	1.6E-18	1.42	[1.31–1.54]	Chen et al. (2013)
Cervical cancer	*HLA-DRB1–HLA-DQA1*	rs9272143	2174/5006	European	9.3E-24	0.67	[0.62–0.72]	Chen et al. (2013)
Cervical cancer	*HLA-DPB2*	rs3117027	2171/4986	European	4.9E-08	1.25	[1.15–1.35]	Chen et al. (2013)

1 OR, odds ratio.

2 *CI, confidence interval*.

Studies of human or *H. pylori* genetics in isolation have generally failed to explain why populations with similar rates of*H. pylori* infection exhibit strikingly different susceptibilities to gastric cancer. For example, in many African and South Asian countries, the low incidences of gastric cancer in the presence of almost universal rates of *H. pylori* infection remain a source of much speculation, and have been referred to collectively as the “African enigma” and the “Asian enigma” (Holcombe, 1992; Campbell et al., 2001; Ghoshal et al., 2007). In Latin America, where *H. pylori* strains native to Amerindian populations have been largely displaced by European strains (Dominguez-Bello et al., 2008; Correa and Piazuelo, 2012), the predominantly Amerindian populations living at high altitudes suffer disproportionately from gastric cancer relative to other populations with similar infection rates (de Sablet et al., 2011; Torres et al., 2013). These and other points of evidence raise the possibility that the pathogenicity of a given *H. pylori* strain may vary with human genomic variation, and that some individuals may be better adapted to their infecting strains than others.

Modern genomic techniques have made the assessment of such hypotheses feasible. Over the past two decades, a comprehensive phylogeography of *H. pylori* has been constructed using multilocus sequence typing (MLST), a procedure by which polymorphisms in fragments from housekeeping genes are used to characterize bacterial isolates (Maiden et al., 1998). Analyses of samples from around the world have revealed a strong concordance between *H. pylori* phylogenetic clusters and the geographical locations from which they are derived (Falush et al., 2003; Moodley and Linz, 2009; Moodley et al., 2009). Ancestral *H. pylori* sequences inferred using MLST data also correspond to geographically defined human populations (Falush et al., 2003; Moodley et al., 2012). The typical modern *H. pylori* chromosome is now understood to be an amalgam of fragments from multiple ancestral sequences, a consequence of *H. pylori’s* high recombinogenicity (Suerbaum et al., 1998; Falush et al., 2003). The genome of an *H. pylori* isolate can thus be quantitatively resolved into ancestral proportions, which correlate with proportions of human ancestry in admixed populations (Kodaman et al., 2014). In some cases, the ancestries of *H. pylori* isolates outperform human mitochondria in differentiating ethnic groups (Wirth et al., 2004).

These shared patterns of ancestry are unlikely to have arisen merely from parallel divergence due to founder effects or neutral drift. Certainly, the well-documented evolvability of functional loci within *H. pylori* strains, even within single individuals over a 6 year span, argues for the importance of adaptive microevolution (Israel et al., 2001; Dorer et al., 2009). Furthermore, at least 25% of known genes, including genes involved in mucosal adherence and the evasion of host immunity, are absent in some*H. pylori* strains isolated from different ethnic groups (Salama et al., 2000; Gressmann et al., 2005). In at least one case, variants of an *H. pylori* gene (*babA2*) encode adhesion proteins that exhibit host-specific effects, a hallmark of co-evolution. BabA binds to blood group antigens, triggering the release of proinflammatory cytokines. Notably, Amerindians, who almost all carry blood group O, harbor strains with a BabA variant that has up to a 1500-fold greater binding affinity to blood group O (Aspholm-Hurtig et al., 2004).

If we conclude from these patterns of genetic covariation that co-evolution between humans and *H. pylori* has occurred and that it has promoted commensalism, then we may ask whether individuals who develop serious clinical disease have inherited mutually ill-adapted sets of host and pathogen alleles. Under this hypothesis, we should expect to find significant interactions between specific pairs of host and pathogen loci in disease models. Toward this end, candidate pairs of loci can be tested based on biochemical evidence of protein–protein interactions, such as those between the adhesin BabA and the Lewis(b) antigen, its epithelial receptor (Backstrom et al., 2004). However, the effect size of any single two-locus interaction may be relatively small, as gastric disease etiology is phenotypically heterogeneous, and likely to be influenced by a large number of human and *H. pylori* genes (El-Omar, 2013). Thus, characterizing the relevant loci in a biologically meaningful way will ultimately require a systems biological approach.

**FIGURE 1:**
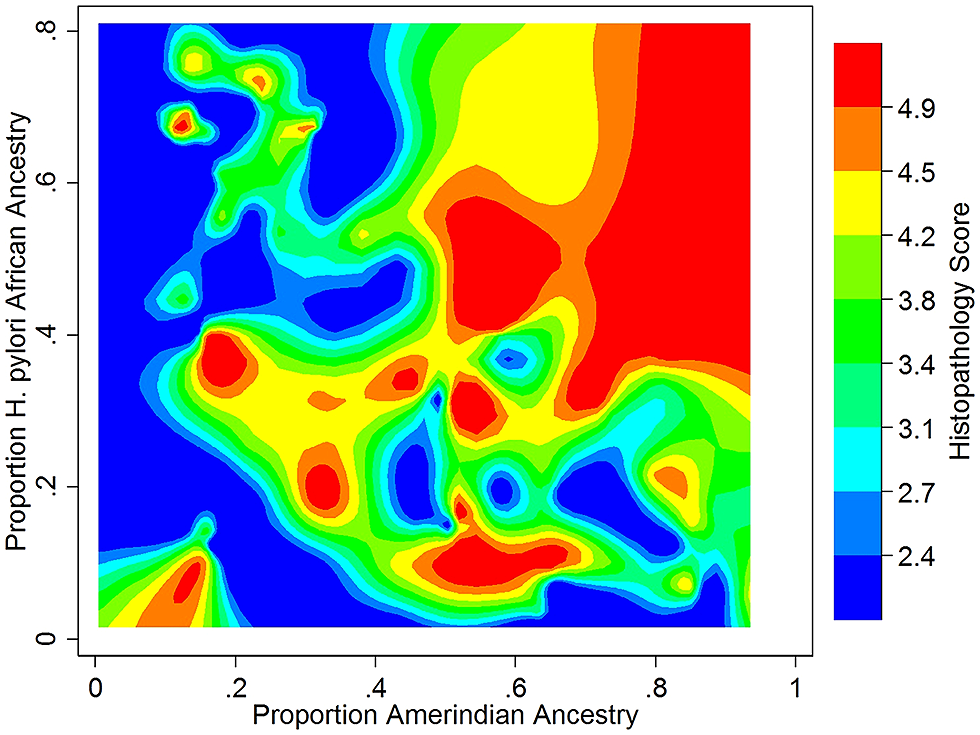
**Gastric histopathology as a function of Amerindian human and African *H. pylori* ancestry in a Colombian population (*N* = 121, age > 39).** Histopathology was scored on a continuous scale, with 2 (blue) representing gastritis and 5 (red) representing dysplasia. Data from Kodaman et al. (2014). Reference samples from the 1000 Genomes Project (Abecasis et al., 2012), HapMap (The International HapMap Consortium, 2005), and the Human Genome Diversity Project (Cavalli-Sforza, 2005) were used to calculate human ancestry, and from the MLST database (Maiden et al., 1998) to calculate *H. pylori* ancestry.

We recently took a broad-based view to assess the impact of human – *H. pylori* co-evolution on gastric disease, using ancestry estimates from both humans and their *H. pylori* isolates in the absence of knowledge of specific interacting loci (Kodaman et al., 2014). Our study participants were recruited from two Colombian populations with highly different rates of gastric cancer, despite a nearly universal prevalence of *H. pylori* infection in both. We found that the low-risk human, coastal population was of admixed African, European, and Amerindian ancestry, whereas the high-risk, Andean population was mainly of Amerindian ancestry, with a minority of European ancestry. Severity of gastric disease correlated with the proportion of African *H. pylori* ancestry in patients with primarily Amerindian ancestry. On the other hand, patients with a large proportion of African human ancestry infected by African *H. pylori* strains had the best prognoses, consistent with ancestral coadaptation, and likely pertinent to the “African enigma.” The interaction between Amerindian human ancestry and African *H. pylori* ancestry accounted for the difference in disease risk between mountain and coastal populations, whereas even the well-known virulence factor, CagA, did not. These findings are thus consistent with the idea that neither human nor *H. pylori* genetic variation confers susceptibility or virulence *per se*, but only in context (**Figure 1**).

These findings also bring to light how understanding co-evolutionary interactions can inform and improve public health measures. It has been suggested that because *H. pylori* dominates the gastric microbiome in infected persons and has been shown to confer some beneficial effects, large-scale antibiotic eradication programs may not be warranted (Bik et al., 2006; Hung and Wong, 2009). Simply estimating ancestry from human samples and *H. pylori* isolates may help to identify individuals at greatest risk for gastric cancer, for whom antibiotic treatment may be most appropriate.

## *MYCOBACTERIUM TUBERCULOSIS* COMPLEX

Another interesting candidate to study from a co-evolutionary perspective is *Mycobacterium tuberculosis* (Mtb) and closely related species, believed to have co-existed with anatomically modern humans for ∼70,000 years (Comas et al., 2013). Since the advent of antibiotics, tuberculosis (TB) has ceased to be as common a cause of human mortality as it once was, but it remains among the most deadly infectious diseases worldwide, with immunocompromised individuals at particularly high risk (Dye and Williams, 2010; Fenner et al., 2013). As with *H. pylori*, the majority of Mtb infections do not develop into clinical disease: 90% of cases are asymptomatic with only latent infection. However, 10% of individuals with latent infections develop TB over their lifetime, for mostly unknown reasons (Barry et al., 2009).

In contrast to *H. pylori,* Mtb is transmitted horizontally, and must cause active disease to be transmitted (e.g., *via* coughing or sneezing). Because Mtb transmission increases with virulence, evolutionary theory predicts that strong selective pressures should favor increased virulence until the number of transmissions per infected host reaches a fitness-reducing limit (Knolle, 1989; Frank and Schmid-Hempel, 2008). Such a limit necessarily depends on population-specific parameters, of which host density is probably the most important (Comas et al., 2013). Thus, the limited pathogenicity and chronicity of Mtb likely reflect its historical adaptation to isolated, low-density human populations. These historical conditions remain relevant in part because Mtb reproduces clonally and without lateral gene transfer; evolution only through point mutations and irreversible gene deletions limits a pathogen’s ability to shift virulence strategies rapidly in response to changing population parameters (Achtman, 2008; Galagan, 2014).

Before advances in genotyping technology improved strain classification, the apparent genetic homogeneity of Mtb led investigators to believe that variation in disease outcome depended primarily on environmental and human genetic factors (Galagan, 2014). Twin and adoption studies provided compelling evidence for the involvement of human genetic variation as a risk modifier (Comstock, 1978). The most recent analyses have calculated the heritable component of Mtb-related immune response phenotypes to range from 30 to 71% (Moller and Hoal, 2010). These findings have motivated a large number of linkage and candidate gene association studies seeking to identify relevant susceptibility loci, but results have often been inconclusive or, worse, contradictory. Many biologically plausible genes, such as those that encode vitamin-D-binding protein (Lewis et al., 2005; Gao et al., 2010), the phagolysomal membrane protein NRAMP/SLC11A1 (Hoal et al., 2004; Velez et al., 2009), and the dendritic adhesion molecule DC-SIGN (Barreiro et al., 2006; Olesen et al., 2007), appear to associate with TB in some human populations, but not others. Inconsistent replication across ethnic groups has also beset the handful of GWAS performed on TB (Chimusa et al., 2014). The few loci that have passed genome-wide significance thresholds also lack clear biological interpretability and fail to explain more than a trivial portion of the estimated heritable component of TB susceptibility (Thye et al., 2010, 2012, **Table 1**).

Since the advent of PCR-based genotyping techniques, it has become increasingly clear that Mtb genetic variation is non-trivial and clinically consequential (Malik and Godfrey-Faussett, 2005; Nicol and Wilkinson, 2008). Most notably, strains now recognized as part of the “Beijing family,” first genotyped in the 1990s following several drug-resistant outbreaks, have been found to exhibit greater efficiency of transmission and to cause more severe disease phenotypes in many animal models (Glynn et al., 2002; Reed et al., 2004; Parwati et al., 2010). Whole-genome sequencing of a large number of clinical Mtb isolates has since revealed over 30,000 Mtb SNPs, a large proportion of which are non-synonymous (Comas et al., 2013; Stucki and Gagneux, 2013). It has been shown that even a few such SNPs can shift a strain from avirulent to virulent (Reiling et al., 2013).

High-throughput sequence data have also enabled the construction of a robust phylogenetic tree, the major branches of which parallel human mitochondrial phylogeny (Comas et al., 2013). Seven major human-adapted Mtb lineages have now been identified, which can be classified as “ancient” or “modern” (Hershberg et al., 2008; Comas et al., 2013). The Beijing family of strains, which causes 50% of infections in East Asia and 13% worldwide, belongs to the most modern lineage. In contrast, *Mycobacterium africanum*, which causes up to half of TB cases in West Africa, belongs to the most ancient Mtb clade, its divergence predating the human migration out of Africa (de Jong et al., 2010). Although strains within all major Mtb lineages induce an overlapping range of immune responses, clade-specific patterns of virulence are emerging. For example, evolutionarily modern lineages appear to induce a less severe early inflammatory response, which possibly increases the efficiency of transmission (Moller and Hoal, 2010; Portevin et al., 2011). A large number of studies in experimental models have also confirmed that diverse Mtb strains reflect substantial functional diversity (Coscolla and Gagneux, 2010).

It is thus likely that genetic factors in both Mtb and humans influence a wide range of TB phenotypes, including those pertaining to infectivity, progression from latent to active disease, and effectiveness of treatment (de Jong et al., 2008; Comas and Gagneux, 2011). However, whether Mtb genetic variation influences disease outcome independently of human genetic variation, and vice versa, is a question that has only recently been addressed (Gagneux, 2012). The mirrored pattern of human and Mtb phylogeography indicates that co-evolution has likely occurred, and consequently, that genome-by-genome interactions may be significant. However, identifying these interactions and assessing their clinical relevance requires the demonstration of heterogeneous outcomes in paired human and Mtb samples of multiple genotypic backgrounds. A small number of published studies to date have met this criterion, assessing previously implicated loci (e.g., in immunogenicity pathways). A study in a Vietnamese cohort found that a variant of the Toll-interleukin 2 receptor (TLR2), known to trigger a cytokine cascade upon recognition of Mtb, increased TB susceptibility only in patients infected with a Beijing strain (Caws et al., 2008). In a Ghanaian cohort, a polymorphism in the immunity-related GTPase M (*IRGM*) gene conferred protection against the European lineage of *M. tuberculosis*, but not *M. africanum* (Intemann et al., 2009). Perhaps of consequence, a gene deletion in the European Mtb strains increases their vulnerability to the autophagy pathway, mediated by IRGM. Thus, the high frequency of the human *IRGM* polymorphism in West Africa has been proposed to explain the competitive advantage of *M. africanum* there (Intemann et al., 2009). The innate immunity-related genes *ALOX5* and *MBL* have also been shown to influence the infectivity of *M. africanum*, but not other strains, in Ghanaian populations (Herb et al., 2008; Thye et al., 2011).

Despite being an ancient strain with ample opportunity to spread beyond West Africa, *M. africanum* has not done so, possibly indicating host-specific adaptation (de Jong et al., 2010; Gagneux, 2012). Other Mtb lineages also appear to associate preferentially with particular human populations, though not as exclusively. A study of ethnically diverse, US-born patients in San Francisco showed that such preferential associations with Mtb lineages persisted even in a cosmopolitan setting (Gagneux et al., 2006). Interestingly, when TB transmission in non-sympatric populations did occur, patients were significantly more likely to be immunocompromised, indicating that non-sympatric Mtb lineages may require some degree of host immunosuppression to compete with sympatric lineages. Mechanisms of Mtb immune evasion, therefore, may have been shaped by population-specific variation in human immune response.

While the above discussion has focused mainly on pulmonary TB, we note here that extra-pulmonary TB, a less common and more severe form of disease, may be especially amenable to analyses guided by co-evolutionary hypotheses. This form of the disease leads more quickly to fatality and results in fewer transmissions than the pulmonary form (Sharma and Mohan, 2004), which probably represents a non-optimal outcome in terms of Mtb fitness. However, data on extra-pulmonary TB to support co-evolutionary hypotheses – especially historical data pre-dating the antibiotic era and the HIV epidemic – are at present lacking (Tiemersma et al., 2011).

## HUMAN PAPILLOMAVIRUS

Human papillomavirus (HPV) is the most common sexually transmitted infectious agent in the world, and the second most common infectious cause of cancer after *H. pylori* (de Martel et al., 2012). Cervical cancer is the major source of mortality associated with HPV, but the virus also causes cancers of the anus, vagina, penis, and oropharynx (zur Hausen, 1989; zur Hausen, 1991; Carter et al., 2001; de Martel et al., 2012). Although over 100 types of papillomaviruses infect humans, only a fraction of them are carcinogenic (Bernard et al., 2010). Infection with two specific types, HPV 16 and HPV 18, account for approximately 70% of cervical cancer cases worldwide, with the remainder of cases largely attributable to 14 other types (Bernard et al., 2010). Nevertheless, the great majority of infections with even carcinogenic HPV types are ultimately benign, demonstrating that HPV infection, although necessary, is not sufficient to cause of cervical cancer (Schiffman et al., 2005; Plummer et al., 2007).

Papillomaviruses (PVs) are notable for their slow rate of evolution relative to other pathogens – only an order of magnitude higher than humans, in the case of HPV (Ong et al., 1993; Rector et al., 2007; Shah et al., 2010). This is commonly attributed to their use of high-fidelity host replication mechanisms (Van Doorslaer, 2013). A slow evolutionary rate precludes rapid adaptation to new hosts, and PV strains correspondingly show little evidence of inter-species transmission or related horizontal gene transfer (Herbst et al., 2009; Shah et al., 2010; Van Doorslaer, 2013). All carcinogenic types of HPV belong to a single genus of papillomaviruses that diverged from a common ancestor about 75 million years ago, predating the primate lineage (Rector et al., 2007; Van Doorslaer, 2013). By the emergence of *H. sapiens*, the common ancestor of HPV 16 and HPV 18 had diverged into separate species, and in fact HPV 16 and HPV 18 had already diverged from all other HPV types within their respective species clades (Lewin, 1993; Ong et al., 1993). Given this combination of early divergence, slow evolution, and strict host specialization, we would expect variants within HPV types independently to have similar phylogeographic patterns to that of *H. sapiens*. Global data collected for the two most frequently sexually transmitted types, HPV 16 and 18, reflect such a pattern (Bernard, 1994). The subtypes and variants of HPV 16 cluster into five major branches of a phylogenetic tree: European (E), Asian/American (AA), East Asian (As), and two African (Af1 and Af2) (Ho et al., 1993; Ong et al., 1993). Subtypes and variants of HPV-18 clustering into three major branches: African (Af), European (E), and Asian + American Indian (As+AI) (Ong et al., 1993).

Biochemical and bioinformatic analyses indicate that HPV evolution has not been entirely neutral. Viral genes expressed early during a PV infection, for example, appear to have evolved at different rates than those expressed late (Garcia-Vallve et al., 2005; Rector et al., 2007). Although most PV genes show signs of strong purifying selection, the exceptions appear to be important (DeFilippis et al., 2002; Chen et al., 2005; Carvajal-Rodriguez, 2008). Two genes under diversifying selection, *E6* and *E7*, are essential for viral replication. They induce cell cycle progression in host cells, and encode proteins that, in the high-risk HPVs, are oncogenic (White et al., 1994; Doorbar, 2006; Klingelhutz and Roman, 2012). Of note, E6 and E7 interfere with the human tumor suppressor proteins, pRB and p53 (Dyson et al., 1989; Huibregtse et al., 1993a,b; Storey et al., 1998; Munger et al., 2004; Doorbar, 2006). In turn, polymorphisms in the human p53 gene were shown to modulate the tumorigenicity of HPV 16 and 18 (Storey et al., 1998). Patients homozygous for the p53Arg mutation were seven times more likely to develop cervical cancer than individuals with 1 or 2 p53Pro alleles (Storey et al., 1998). Other human polymorphisms, such as those in the genes *RPS* and *TYMS*, influence HPV transmissibility. In a study of high-risk HPV infections in Nigerian women, variants in these genes were shown to modulate risk of infection with HPV 16 and 18. Despite the effects described above, genetic variation in neither the host nor the pathogen has been successful in explaining most heritable risk of HPV-associated disease, when considered in isolation (Magnusson et al., 2000; Hildesheim and Wang, 2002; Wheeler, 2008; Chen et al., 2013; Shi et al., 2013, **Table 1**).

Because the integration of the HPV genome within the human genome is permanent, death of the host ends all possibility of viral multiplication and transmission. Even strains that damage the health of the host sufficiently to reduce human-to-human sexual contact can suffer a competitive disadvantage. Therefore, both host and pathogen should cooperate to prevent severe disease. As with *H. pylori* and MTB, there is some empirical evidence supporting the idea that humans and HPV types co-evolved to limit tumorigenesis, and that evolutionarily mismatched strains may be driving severe clinical outcomes. A study of high-grade cervical intraepithelial neoplasia (CIN) and invasive cervical cancer in an Italian cohort of Caucasian women demonstrated that non-European variants of HPV16, Af1 and AA, were found at an increased frequency in invasive lesions (Tornesello et al., 2004). A separate study of mostly Caucasian (81%) female university students in the United States showed that those infected with non-European HPV 16 variants were 6.5 times more likely to develop high-grade CIN than those with European variants (Xi et al., 1997). The same study demonstrated a similar HPV 16-related risk profile (4.5 relative risk) in a predominantly Caucasian (79%) population of women presenting at a sexually transmitted disease clinic (Xi et al., 1997). Finally, at the molecular level, there is some evidence that variants of the HPV 16 E6 protein, described above, may be better adapted for replication within specific hosts (DeFilippis et al., 2002).

## DISCUSSION

Taken together, the three examples above illustrate how co-evolution can promote a reduction in antagonism between pathogen and host, and in doing so leave discernible signatures on the genomes of both species. If, as we argue here, the disruption of historical co-evolutionary relationships can explain many differences in disease outcomes, knowledge of the conditions under which such relationships arise and dissolve will be helpful in defining genetic architecture of disease etiology. The applicability of this model depends, to a large extent, on the degree of integration between host and pathogen genomes, which can take many forms.

A long-standing association between humans and pathogens may be a necessary factor for cross-genomic integration, as with the three pathogens we have discussed. In contrast, many infectious diseases that occur epidemically are caused by zoonotic pathogens for which the human host is an evolutionary dead end, such as *Salmonella enterica* and *Borrelia burgdorferi* (Sokurenko et al., 2006; Falush, 2009). Other pathogens have had limited occasion to co-evolve with humans, because they cause disease primarily on an opportunistic basis (e.g., *Streptococcus pneumonia* or *Clostridium difficile*) or over a broad range of hosts (e.g., *Toxoplasma gondii*) (Ajzenberg et al., 2004; Sokurenko et al., 2006). The epidemic outbreaks caused by these pathogens may leave detectable signatures on the human genome, but reciprocal evolution in the pathogen need not occur.

For human-specific pathogens that cause endemic diseases and are not recent, the likelihood that severe disease is the outcome of a co-evolutionary mismatch should increase with the overlap between host and pathogen fitness. The pathogenicity of vertically transmitted pathogens, for example, should decrease over time, because such pathogens often depend on host survival (and possibly reproduction) for transmission. However, a strong overlap between host and pathogen fitness can also exist in the absence of vertical transmission. A horizontally transmitted pathogen, such as HPV, can evolve to be largely benign insofar as it depends on a healthy host for transmission.

When a pathogen’s fitness depends on its ability to cause damage to its human host, as with Mtb, attenuated antagonism becomes a special case, and its disruption becomes more difficult to detect and requires more evidence to confirm. While Mtb strains that increase the duration of a transmissible state will generally have a competitive advantage, the optimal duration can be expected to vary based on many population-level parameters, such as host density. This probably explains why modern Mtb lineages that are more common in high-density urban populations exhibit greater virulence. On the other hand, if horizontal transfer is confined to small, isolated populations, it may be considered effectively vertical. With such pathogens, a better understanding of the co-evolutionary history will be necessary to infer whether severe disease is caused by disrupted co-evolution or by another factor, such as infection by a universally more virulent strain or an opportunistic infection in an immunosuppressed patient.

The life history of the pathogen is also important in assessing the possibility and nature of co-evolution. A pathogen typically faces a tradeoff between fecundity and longevity. Increased fecundity within a host increases the probability (or rate) of transmission, but may negatively affect host lifespan or mobility (Frank and Schmid-Hempel, 2008). Therefore, a pathogen’s position on the continuum between greater fecundity and increased longevity will often reflect the degree to which its fitness depends on the health of the host. The case of HPV is somewhat of an exception in this regard. Host immune responses can induce diverse strategies, creating HPV types that are highly fecund, or less fecund with few virions per host. Whereas highly fecund types are more likely to transmit, they are also more likely to induce a vigorous immune response leading to clearance. Low fecundity types on the other hand, are more likely to persist as subclinical infections that can lead to prolonged inflammation and eventually cancer (DeFilippis et al., 2002). However, human populations that co-evolved with specific variants of these persistent types may be less likely to develop cancer, as described above.

Another factor influencing the applicability of the model we propose is a pathogen’s recombinogenicity. In theory, a pathogen that recombines freely is more likely to be panmictic, and hence less likely to co-evolve with a particular human host population (Bull et al., 1991). In fact, epidemic disease outbreaks often follow recombination events, and the pathogens responsible for the epidemics often appear superficially clonal, likely reflecting the rapid proliferation of especially successful recombinant strains (Grigg et al., 2001; Heitman, 2006). A case in point is *Neisseria meningitides* (Falush, 2009), as well as the eukaryotic parasites *Toxoplasma gondii* and*Plasmodium falciparum*, which though able to recombine sexually, exhibit surprisingly limited genetic diversity (Grigg et al., 2001). On the other hand, the strict clonality of Mtb and HPV has likely favored co-evolution, leading to reduced antagonism, while recombination in *H. pylori* can disrupt the co-evolutionary relationship favored by vertical transmission.

Recombination can also occur via horizontal gene transfer, as among species within the microbiome (Smillie et al., 2011; Ravel et al., 2011; Liu et al., 2012). This would suggest that co-evolution might be a relatively weak force in shaping microbiotal genetic variation. However, data possibly supporting human–microbiome co-evolution exist; for example, the strongest correlate of an individual’s microbiotal identity is ethnicity (Benson et al., 2010; Human Microbiome Project Consortium, 2012). The extent to which this correlation is driven by mutual genetic factors is unclear, as recurring environmental exposure and frequent vertical transmission may also account for most, if not all of it (Turnbaugh et al., 2009). Assessing whether the genomes of the microbiome and humans are integrated will be a key area of research, as it relates to co-evolution and disease risk (McFall-Ngai et al., 2013).

## CONCLUSION

While the prospect of introducing co-evolutionary interactions into genetic epidemiology models may appear to add a new layer of complexity to an already difficult problem, a co-evolutionary perspective should help us construct more precise and accurate hypotheses, improving our ability to find real and reproducible results. Importantly, co-evolved genes will not be neutral in either species, which may make their identification easier. Although many methods exist to find loci that are candidates to have evolved under selection (Aguileta et al., 2009; Karlsson et al., 2014), and these methods can assess the strength, timing, and direction of selection (e.g., balancing or positive), they are not at present well adapted to the study of joint patterns of selection.

If the ultimate goal is to find interacting genes that have co-evolved to be benign and are subsequently disrupted in disease, we will need to identify differential patterns of concerted selection in paired human and pathogenic loci from different populations. The limiting factor to the development of appropriate methods toward this end has probably been the lack of prospectively collected paired genetic data for humans and pathogens. Once these data are available, existing methods to detect epistasis within a species can be adapted for cross-species analyses in the absence of *a priori* biological hypotheses. Where evidence for selection exists, genetic variants can be filtered prior to analyses to detect epistasis. Framing hypotheses in the context of biochemical and bioinformatic functional evidence or pre-existing evidence for association can hone study design even further. For example, using paired data and pathogenic genetic variation as the outcome variable, novel epitopes have been discovered in association studies (Bartha et al., 2013). Such data can be used to mitigate the immense multiple testing burden incurred by a hypothesis-free approach to detecting genetic interactions.

Finally, we should note that the ultimate impact of this approach may extend beyond infectious diseases to what are traditionally considered non-communicable diseases. For example, we now recognize that both gastric and cervical cancers, as well as atherosclerosis, may have origins in infection (Libby et al., 2002; Porta et al., 2011). The number of such examples will certainly expand.

## Conflict of Interest Statement

The authors declare that the research was conducted in the absence of any commercial or financial relationships that could be construed as a potential conflict of interest.
